# Effect of intravenous different drugs on the prevention of restlessness during recovery period of pediatric laparoscopic surgery: a randomized control trial

**DOI:** 10.1007/s00540-024-03410-9

**Published:** 2024-09-29

**Authors:** Zhi-Jie Liang, Jia-Mei Liang, Xiao-Ling Nong, Ni-Qiao Chen, An-Yuan Liu, Xiao-Qiang Sun, Yi-Xing Lu, Zhuo-Xin Ou, Sheng-Lan Li, Yu-Nan Lin

**Affiliations:** 1 Department of Anesthesiology, Guangxi Key Laboratory of Enhanced Recovery after Surgery for Gastrointestinal Cancer, the First Affiliated Hospital of Guangxi Medical University, NO.6 Shuangyong Road, Nanning, 530021 China; 2 Guangxi Medical University, Nanning, China; 3 Department of Anesthesiology, Sichuan Province Orthopedic Hospital, Chengdu, China; 4 Department of Anesthesiology, Maternity and Child Health Care of Guangxi Zhuang Autonomous Region, Nanning, China

**Keywords:** Emergence agitation, Dexmedetomidine, Esketamine, Laparoscopic surgery in children

## Abstract

**Purpose:**

To explored the impact of dexmedetomidine and esketamine in mitigating restlessness during the postoperative recovery phase following laparoscopic surgery in children.

**Methods:**

102 individuals aged 1 to 7 years experiencing laparoscopic surgery were randomly allocated into three groups, each accepting 1 μg/kg of dexmedetomidine, 0.3 mg/kg of esketamine, or saline immediately at the end of carbon dioxide pneumoperitoneum. Emergence agitation (EA) occurrence was assessed by PAED scale and 5-point agitation scale. Pain was judged using Face, Legs, Activity, Cry, and Consolability (FLACC) scale. The recovery time, extubation time, and post-anesthesia care unit (PACU) stay time were recorded for all three groups.

**Results:**

Patients administered 1 μg/kg of dexmedetomidine (8.8%) and individuals given 0.3 mg/kg of esketamine (11.8%) showed lower incidences of emergence agitation compared to those receiving saline (35.5%; *P* = 0.009). There was no statistically significant difference in the time to discharge from the PACU among the three groups of patients (*P* > 0.05). The recovery time and extubation time were notably extended in the dexmedetomidine group (40.88 ± 12.95 min, 42.50 ± 13.38 min) when compared to the saline group (32.56 ± 13.05 min, 33.29 ± 11.30 min; *P* = 0.009, *P* = 0.010).

**Conclusion:**

Following CO_2_ pneumoperitoneum in pediatric laparoscopic surgeries, the intravenous administration of 1 μg/kg dexmedetomidine or 0.3 mg/kg esketamine effectively lowers EA occurrence without extending PACU time.

## Introduction

Emergence agitation (EA) can occur in individuals of any age, especially in preschool children. The concept of post-anesthetic restlessness was first introduced by Eckenhoff in 1960 [1]. With the deepening of cognition, Sikich et al. [2] defined it as a cognitive and behavioral disorder following general anesthesia, often presenting as groaning, anxiety, and involuntary tossing and turning in bed. EA typically arises within the initial half an hour post-anesthesia, with its occurrence ranging from 10 to 80% [3]. With the advancement of endoscopic technology, an increasing number of children are undergoing laparoscopic surgery under general anesthesia. Relevant studies have indicated that the establishment of pneumoperitoneum during laparoscopic surgery can lead to an average 3% reduction in cerebral oxygen saturation in children. The decrease in cerebral oxygen saturation might have an impact on the restoration of consciousness in patients undergoing general anesthesia [4, 5]. Literature has once reported that the incidence of EA in pediatric laparoscopic surgery can even reach as high as 45.8% [6]. Currently, there are relatively few related studies on how to prevent the occurrence of EA in pediatric laparoscopic surgery. EA not only poses a risk of patients’ self-injury, but also places additional strain on medical staff and reduces the satisfaction of patients’ families with anesthesia.

Currently, drugs such as opioids, midazolam, propofol, esketamine, and dexmedetomidine are frequently utilized in clinical settings to prevent and manage EA. Nevertheless, opioids (respiratory suppression), midazolam (drug accumulation and prolonged recovery time), and propofol (inhibition of respiration and circulation, injection pain) are not very ideal for application due to the side effects inherent in these drugs. Dexmedetomidine exhibits sedative, hypnotic, analgesic, anti-sympathetic effects, etc. Within a safe dosage range, it does not suppress respiration and induces sedation similar to natural sleep, facilitating easy awakening. Hence, it is extensively employed for preoperative sedation in pediatric patients [7]. Esketamine exerts calming, hypnotic, analgesic, and antidepressant impacts by inhibiting N-methyl-D-aspartic acid receptor (NMDA), which can be widely used for perioperative sedation in children [8, 9].

This study explored the effects of preventive administration of dexmedetomidine and esketamine at the end of pediatric laparoscopic surgery on EA, and observed the occurrence rate of EA in children patients, the changes of heart rate (HR) and pulse oxygen saturation (SpO_2_), and the quality of awakening. We aimed to provide clinical evidence supporting the reduction of agitation incidence during the recovery phase in children undergoing laparoscopic surgery.

## Methods

### Study design and patient selection

This prospective, randomized, placebo controlled, single-site trial was carried out between December 2022 and February 2023 at the First Affiliated Hospital of Guangxi Medical University. This study was approved by the Ethics Committee of the First Affiliated Hospital of Guangxi Medical University (NO. 2022-K067-01, October 14, 2022) and registered with the Chinese Clinical Trial Registry (NO. ChiCTR2200066542, December 8, 2022). Informed consents were secured. The design and implementation of this study are based on the Consolidated Standards of Reporting Trials guidelines.

Inclusion criteria: aged 1–7 years, weight < 30 kg, BMI of 12–24 kg/m^2^, children who were categorized as ASA-PS 1 or 2. Exclusion criteria: developmental impairment, neurological condition, cardiovascular disorder, recent intake of sedatives or pain-relieving medications, known allergies to the study drugs, or parental refusal.

### Anesthesia and interventions

**Fig. 1:**
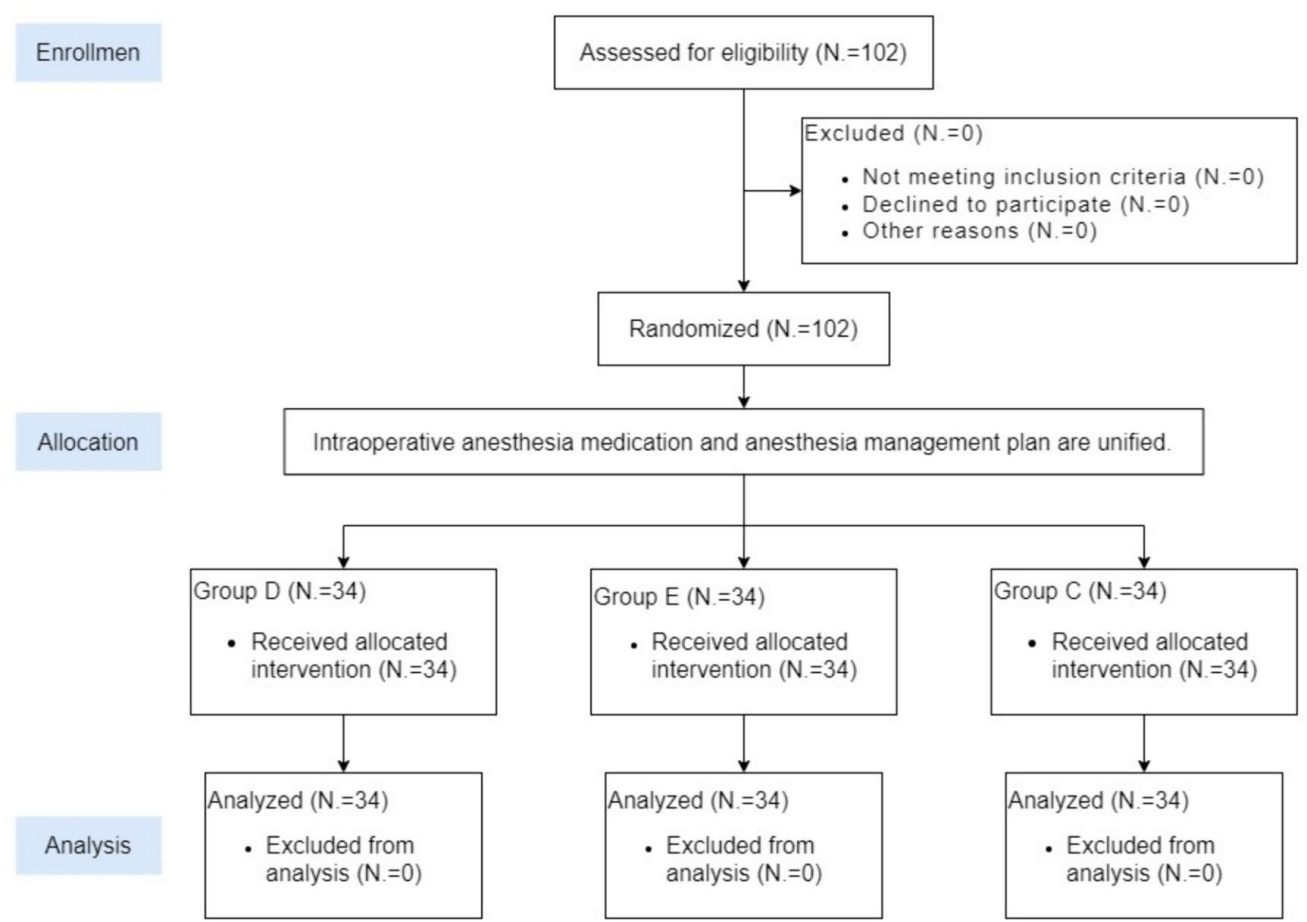
Flow chart

102 participants were assigned randomly to one of three groups. At the end of CO_2_ pneumoperitoneum, Control group (group C) was administered 10 ml of normal saline; Dexmedetomidine group (group D) was given 1 μg/kg of dexmedetomidine [10], which was diluted to 10 ml with normal saline; Esketamine group (group E) was administered 0.3 mg/kg of esketamine [11], which was diluted to 10 ml with normal saline. The drugs in all three groups were intravenously pumped for 10 min. To ensure blinding, researchers who did not participate in the previous process registered and randomly assigned patients Fig. 1.

Premedication was not administered. A guardian or nurse went with pediatric patient to the operating theater. All patients were required to fast for 8 h. Before entering the operating room, midazolam (0.1 mg/kg) was administered intravenously in the anesthesia holding area. Vital signs were continuously monitored throughout the entire surgical process.

Patients received IV induction with propofol (2.5 mg/kg) and remifentanil (2 μg/kg) followed by maintenance with propofol (9–12 mg/kg/h) and remifentanil (0.2–0.4 μg/kg/min). Cisatracurium (0.2 mg/kg) was administered for intubation. After tracheal intubation was completed, all patients in the three groups were given 0.25% ropivacaine under ultrasound guidance for bilateral transversus abdominis plane block (TAPB) to prevent postoperative pain. Anesthesia was maintained using volume-controlled ventilation with a goal end-tidal CO_2_ of 35-45 mmHg. Immediately after carbon dioxide pneumoperitoneum, patients were given intravenous fentanyl (2 μg/kg) and tropisetron (0.1 mg/kg) for postoperative analgesia and preventive antiemesis.

After the surgery, the children were transferred to the PACU for anesthesia recovery. The ventilator was used to maintain breathing control in accordance with the intraoperative respiratory parameters, while noninvasive blood pressure, pulse oxygen saturation, and heart rate were monitored. When the children regained spontaneous breathing, became fully awake, and recovered the swallowing and choking reflexes, and the tidal volume was greater than 6 ml/kg, the tracheal tube could be extubated. After extubation, the children were observed in the PACU for at least 30 min. A 5-point agitation scale [12] and Pediatric Anesthesia Emergence Delirium (PAED) scale [2] were utilized for emergency arousal evaluation, with scores recorded at 0, 5, and 30 min post-tracheal tube removal. The score of PAED scale ≥ 12 or 5-point agitation scale ≥ 4 was indicated a diagnosis of EA. IV propofol 1 mg/kg was employed to manage EA when child could not be comforted. Pain levels were assessed utilizing the Face, Legs, Activity, Cry, and Consolability (FLACC) scale [13]. Individuals were released from PACU once they achieved a Aldrete score[14] of 9.

### Outcomes measures

The main outcome variable was the incidence of EA. Evaluations were conducted by other researchers for each patient in the PACU.

The secondary outcomes comprised recovery time (the time between withdrawal of anesthetic drugs and awakening upon call), extubation time (the time between withdrawal of anesthetic drugs and extubation), and PACU stay time (the time span from entering the PACU to departing from it). HR and SpO_2_ were documented at several time intervals: before administration (T_0_), immediately post-administration (T_1_), during the PACU stay (T_2_), at extubation (T_3_), 5 min post-extubation (T_4_), and 30 min post-extubation (T_5_). Adverse events such as bradycardia (the HR was less than 20% of the baseline value), respiratory depression, hypoxemia (the SpO_2_ was below 90%), laryngeal spasm, nausea, vomiting, and drowsiness were monitored during anesthesia, recovery period, and 1 h after returning to the ward.

## Statistical analysis

A sample size calculation was performed using PASS software (Version 15.0; NCSS, USA). In this study, the primary outcome was the incidence of EA. Based on our preliminary results, the incidence of EA was 40% (6 of 15) in group C, 6.7% (1 of 15) in group D, and 13.3% (2 of 15) in group E. A priori power analysis using two-sided analysis with an α error of 0.05 and a power (1-β) of 0.8 showed that 25 patients were needed to detect a statistical difference in the incidence of EA between the three groups for this study. The sample size was increased to 34 to allow for dropout in each group.

Performed statistical analysis was performed using SPSS 25.0. *P* < 0.05 was statistically meaningful. Normally distributed continuous variables: mean ± SD (one-way ANOVA). Non-normally distributed continuous data: medians and interquartile ranges (Kruskal–Wallis test). Categorical variables: numbers and percentages (Chi-square test or Fisher’s exact test as appropriate). Multiple comparisons: post hoc with adjusted alpha.

## Results

102 individuals were admitted into this study, all of whom underwent surgery, anesthesia, and data collection. The three groups comprised 43 cases of oblique inguinal hernia, 52 cases of hydrocele, and 7 cases of cryptorchidism. The distribution was 13:19:2 in group C, 16:15:2 in group D, and 14:18:3 in group E. No notable disparity in basic information was observed among these groups (Table 1).

**Table 1:** Patient characteristics

Variable	Group D (*n* = 34)	Group E (*n* = 34)	Group C (*n* = 34)	*P*
Age (years)	3 (2,5)	3(2,6)	4 (2,5)	0.539
Sex (male/female)	30/4	30/4	29/5	0.916
Height (cm)	97.5 (84.5,108.5)	103 (91.5,123.5)	105 (90.0,113.5)	0.158
Weight (kg)	15.25 ± 3.77	17.40 ± 5.17	16.35 ± 4.80	0.165
BMI (kg/m^2^)	15.43 (13.97, 17.32)	15.59 (13.83, 16.85)	14.95 (14.18, 16.33)	0.985

*BMI*: body mass index. Data are presented as mean ± SD or median (interquartile range)

The incidence of EA was 8.8% in group D, 11.8% in group E, and 35.5% in group C. The variations between the groups displayed statistical significance (*P* = 0.009 each; Table 2). Surgical procedure duration and PACU discharge time were comparable among these groups (*P* > 0.05). Nevertheless, group D experienced prolonged awakening and extubation times compared to group C (Table 2).

**Table 2:** Incidence of EA, recovery time, extubation time, duration of surgery, and PACU stay time

Variable	Group D (*n* = 34)	Group E (*n* = 34)	Group C (*n* = 34)	*P*
Incidence of EA	3 (8.8%)^d^	4 (11.8%)^d^	12 (35.3%)	0.009
Recovery time (min)^a^	40.88 ± 12.95^d^	35.94 ± 12.24	32.56 ± 13.05	0.009
Extubation time (min)^b^	42.50 ± 13.38^d^	38.47 ± 11.81	33.29 ± 11.30	0.010
Duration of surgery (min)	41.94 ± 15.73	46.94 ± 17.51	43.53 ± 16.94	0.456
PACU stay time (min)^c^	88.71 ± 11.02	82.59 ± 17.74	81.88 ± 17.53	0.150

Data are presented as mean ± SD or number (%)

*EA*: emergence agitation; *PACU*: post-anesthesia care unit

^a^ The time between withdrawal of anesthetic drugs and awakening upon call

^b^ The time between withdrawal of anesthetic drugs and extubation

^c^ The time span from entering the PACU to departing from it

^d^ *P* < 0.05 compared with group C

Dramatically statistical differences were observed in the agitation scores at T_3_, T_4_, and T_5_, as well as PAED and FLACC scores among groups (*P* < 0.05). Relative to group C, group D exhibited lower agitation scores at T_3_, T_4_, and T_5_, as well as a lower PAED scale (*P* < 0.05). FLACC score at T_4_ for group D was found to be lower in comparison to group C (*P* < 0.05). Group E displayed reduced agitation scores at T_5_ and lower FLACC scores at T_3_, T_4_, and T_5_ relative to group C (*P* < 0.05) (Table 3).

**Table 3:** The 5-point agitation scale, PAED scale, and FLACC scale

Variable	Group D (n = 34)	Group E (n = 34)	Group C (n = 34)	*P*
The 5-point agitation scale				
T_3_	1 (1,3)^a^	3 (1,3)	3 (1.75,4)	0.024
T_4_	1 (1,3)^a^	2 (1,3)	3 (1,4)	0.001
T_5_	1 (1,3)^a^	2 (1,2)^a^	3 (2,4)	0.001
PAED scale				
T_3_	4 (3,10)^a^	8 (4,10)	10 (4,12.75)	0.028
T_4_	4 (2,10)^a^	5 (3.5,10)	10 (4,13)	0.006
T_5_	3.5 (2,10)^a^	5 (3.5,8.25)	10 (4,13)	0.006
FLACC scale				
T_3_	5 (5,6.5)	5 (3,10)^a^	8 (5,10)	0.006
T_4_	5 (3,6)^a^	5 (3,7.5)^a^	8 (5,10)	0.001
T_5_	5 (3,6)	3 (2,6)^a^	5 (5,10)	0.006

Data are presented as median (interquartile range)

*PAED scale*: Pediatric Anesthesia Emergence Delirium scale; *FLACC scale*: Face Legs, Activity, Cry, and Consolability behavioral pain assessment tool; *T*_*3*_: at extubation; *T*_*4*_: 5 min post-extubation; *T*_*5*_: 30 min post-extubation

^a^ *P* < 0.05 compared with group C

Statistical differences in HR at T_1_, T_3_, T_4_, and T_5_ were observed among groups (*P* < 0.05). No notable differences in SpO_2_ were noted among these groups at six time points (Table 4).

**Table 4:** HR and SpO_2_ of the three groups at each time point

Variable	Time	Group D (*n* = 34)	Group E (*n* = 34)	Group C (*n* = 34)	*P*
HR (bpm)	T_0_	94.53 ± 14.79	90.56 ± 12.04	96.76 ± 15.10	0.187
	T_1_	85.06 ± 15.79^ab^	89.00 ± 11.31	94.12 ± 14.06	0.029
	T_2_	91.47 ± 12.92	85.94 ± 13.47^a^	88.56 ± 14.54^a^	0.253
	T_3_	113.76 ± 19.71^ab^	114.00 ± 20.31^ab^	125.68 ± 19.40^a^	0.021
	T_4_	101.62 ± 18.88^ab^	109.59 ± 12.03^a^	116.12 ± 20.02^a^	0.012
	T_5_	95.29 ± 15.74^b^	107.65 ± 12.10^ac^	104.82 ± 16.91^a^	0.003
SpO_2_ (%)	T_0_	99.94 ± 0.34	99.85 ± 0.50	99.91 ± 0.38	0.670
	T_1_	99.91 ± 0.29	99.97 ± 0.03	99.88 ± 0.32	0.394
	T_2_	99.53 ± 1.10	99.76 ± 0.61	99.68 ± 0.77	0.519
	T_3_	98.94 ± 1.39^a^	98.56 ± 1.91^a^	99.15 ± 1.26^a^	0.333
	T_4_	99.29 ± 0.94^a^	99.38 ± 0.85^a^	99.26 ± 1.54^a^	0.909
	T_5_	99.44 ± 0.96^a^	99.38 ± 0.99^a^	99.56 ± 0.66^a^	0.703

Data are presented as mean ± SD

*HR*: heart rate; *SpO*_*2*_: pulse oxygen saturation; *T*_*0*_: before administration; *T*_*1*_: immediately post-administration; *T*_*2*_: during the PACU stay; *T*_*3*_: at extubation; *T*_*4*_: 5 min post-extubation; *T*_*5*_: 30 min post-extubation

^*a*^*P* < 0.05 compared with T_0_; ^*b*^*P* < 0.05 compared with group C; ^*c*^*P* < 0.05 compared with group D

The highest occurrence of bradycardia was observed in group D at 14.7%, followed by group E at 2.9%, with no occurrences in group C. No respiratory depression, hypoxemia, laryngeal spasm, nausea, vomiting, or drowsiness were observed in any of the three groups (Table 5).

**Table 5:** Postoperative adverse events

Variable	Group D (*n* = 34)	Group E (*n* = 34)	Group C (*n* = 34)	*P*
Bradycardia^b^	5 (14.7%)^a^	1 (2.9%)	0	0.045
Respiratory	0	0	0	–
Depression	0	0	0	–
Hypoxemia^c^	0	0	0	–
Laryngeal spasm	0	0	0	–
Nausea and vomiting	0	0	0	–
Drowsiness	0	0	0	–

Data are presented as number (%)

^a^ Group D vs. group C (*P* = 0.027), group E vs. group C (*P* = 0.05), group D vs. group E (*P* = 0.099)

^b^The HR was less than 20% of the baseline value

^c^The SpO_2_ was below 90%

## Discussion

Oblique inguinal hernia and hydrocele rank among the most prevalent surgical disorders in kids, with reported incidences ranging from 0.8% to 4.4% in literature. Male children experience these conditions at a rate 5 to 10 times higher than female children [15–17]. At present, laparoscopy has become the main method for inguinal hernia repair. Laparoscopic hernia repair offers advantages such as reduced trauma, shorter hospital stay, quicker recovery, and easier detection of contralateral hernia compared to traditional open repair [18, 19]. It has been widely adopted in pediatric surgery in recent decades. However, due to the immature development of children’s cognitive function, they cannot cooperate with the surgery and local anesthesia, nerve block anesthesia, intraspinal anesthesia and other anesthesia methods cannot be used. Therefore, general anesthesia is often adopted for pediatric anesthesia. EA often occurs in children after pediatric laparoscopic surgery. The pathogenesis of EA remains ambiguous. At present, the widely acknowledged risk factors inducing EA mainly consist of: (1) age, (2) preoperative anxiety, (3) utilization of inhaled anesthetics, (4) type of surgery, and (5) exposure to undesirable stimuli [20–23]. Research indicates that agitation during the recovery phase of laparoscopic surgery in children can occur at a rate as high as 45.8% [6], and the reasons are considered to be hypoxemia, hypercapnia, pain, hypothermia, etc. [24].

Dexmedetomidine provides sedative, hypnotic, anti-anxiety, sympathetic inhibition, analgesic, and other effects, and can reduce the release of endogenous catecholamines and weaken stress response, thus maintaining hemodynamic stability [25, 26]. Increased doses of dexmedetomidine did not inhibit breathing and induced sedation similar to the natural sleep state, facilitating easy awakening. Esketamine exhibits 3–4 times greater affinity for NMDA receptors than its R-enantiomers, allowing it to produce sedative, hypnotic, analgesic, and antidepressant effects at low doses [27]. Researches suggest that IV administration of dexmedetomidine at doses ranging from 0.21 to 1.00 μg/kg [28] and esketamine at doses from 0.3 to 0.7 mg/kg [29] effectively reduce the incidence of EA after surgery. In this study, the incidence of agitation was the highest in group C (35.3%), followed by group E (11.8%), and the lowest in group D (9.1%). The use of dexmedetomidine and esketamine can both effectively reduce the incidence of EA, which is consistent with the conclusions of Xin et al. [30] Our findings indicated that IV pump injection of dexmedetomidine and esketamine could effectively apply laparoscopic surgery in children, with no observed adverse reactions such as respiratory depression and hypoxemia. This approach proved beneficial in reducing agitation during the postoperative recovery period in kids.

Low doses of ketamine can decrease EA occurrence without prolonging recovery and extubation time [31], aligning with our findings. After IV injection, dexmedetomidine takes effect within 15 min, with sedation lasting 105–225 min, a 6-min distribution half-life, and a 1.8-h clearance half-life [32]. The recovery and extubation time in group D were 40.88 ± 12.95 min and 42.50 ± 13.38 min, which were exceeding that in group C, indicating that dexmedetomidine prolonged both the recovery and extubation time. However, a meta-analysis concluded that dexmedetomidine had no effect on the recovery time and extubation time. This outcome could be influenced by factors such as sample size, administration time, and dosage [33].

In the cardiovascular system, dexmedetomidine acts on presynaptic α-2 adrenergic receptors to inhibit the release of catecholamines by the sympathetic nerves. This action leads to a gradual increase in vagal tone, bradycardia, hypotension, and other manifestations in some cases [34, 35]. Studies have demonstrated that IV infusion of 0.66 μg/kg and 1 μg/kg dexmedetomidine within 10 min can result in approximately 25% and 15% reductions in systolic BP and HR, respectively [36]. The HR at T_0_ and T_1_ in group D was 94.67 ± 15.00 bpm and 85.06 ± 16.035 bpm, respectively. This indicated that IV pump injection of dexmedetomidine could significantly decrease HR, which was related to the hemodynamic effect of dexmedetomidine and infusion speed and dose [37]. In group E, the HR at T_0_ was 90.56 ± 12.04 bpm, which did not change significantly compared to 89.00 ± 11.31 bpm after administration of esketamine. This is consistent with the research results of Austin et al. [38] and may be related to the low-dose administration of esketamine. Esketamine has mental side effects such as hallucinations, nightmares, restlessness, anxiety, and disorientation. All the children in this study were able to tolerate esketamine, and no occurrence of the above mental symptoms was observed. This is consistent with the research conclusion of Li et al. [39]. Because the affinity of esketamine to the NMDA receptor is 3 to 4 times that of the levorotatory enantiomer, a small dose of esketamine can produce anesthesia and analgesia, and the recovery time is faster. Therefore, it may reduce dissociative and psychotomimetic side effects to a certain extent [27].

## Conclusion

Prophylactic dexmedetomidine administration at 1 μg/kg and esketamine at 0.3 mg/kg has been shown to safely and effectively reduce EA occurrence in kids. IV pumping of dexmedetomidine may lead to bradycardia, but it results in more stable HR fluctuations after extubation, prolongs emergence time and extubation time, without extending the PACU stay. The occurrence of adverse reactions of IV pump esketamine is low, and it does not extend the emergence time, extubation time, and PACU stay time.

## Data Availability

The data that support the findings of this study are available from the corresponding author upon reasonable request.
